# The Two-Way Polyphenols-Microbiota Interactions and Their Effects on Obesity and Related Metabolic Diseases

**DOI:** 10.3389/fnut.2019.00188

**Published:** 2019-12-20

**Authors:** Telma Angelina Faraldo Corrêa, Marcelo Macedo Rogero, Neuza Mariko Aymoto Hassimotto, Franco Maria Lajolo

**Affiliations:** ^1^Department of Food Science and Experimental Nutrition, School of Pharmaceutical Sciences, University of São Paulo, São Paulo, Brazil; ^2^Food Research Center, CEPID-FAPESP (Research Innovation and Dissemination Centers, São Paulo Research Foundation), São Paulo, Brazil; ^3^Department of Nutrition, School of Public Health, University of São Paulo, São Paulo, Brazil

**Keywords:** polyphenols, obesity, inflammation, microbiome, metabolism

## Abstract

Metabolic diseases can change the gut microbiota composition and function, and pathogenic bacteria contribute to the development of metabolic disorders. Polyphenols may act in the gut microbiota to favor the increase of beneficial bacteria and hamper the increase of pathogenic bacteria. In addition, the microbiota may act on polyphenols to increase their bioavailability. This two-way interactions between polyphenols and the gut microbiota could affect human metabolism and reduce cardiometabolic risk. Despite the possible benefits of polyphenols for human health through modulating the microbiome, studies are scarce, and present several limitations. This review provides an overview of the polyphenol–microbiota interactions and its effects on metabolic disorders.

## Introduction

Obesity is associated with other metabolic diseases including type 2 diabetes (T2D), metabolic syndrome, cardiovascular diseases (CVD), non-alcoholic fatty liver disease (NAFLD), and certain cancers (1). White adipose tissue (WAT) regulate energy homeostasis through the secretion of hormones, adipokines, and growth factors. WAT enlargement causes the recruitment of macrophages and other immune cells into the WAT. This low-grade inflammation disrupts metabolic processes, resulting in impaired glucose, and fatty-acid uptake and metabolism, thereby contributing to the development of metabolic disease (2, 3). In this context, an increased inflammatory response is closed related to the insulin resistance in obese subjects. This response activates Toll pathways kinases and tumor necrosis factor alpha (TNF-α) receptors such as c-jun N-terminal kinase (JNK)-1, and inhibitor of kappa B kinase (IKK). These kinases can phosphorylate the insulin receptor substrate (IRS)-1 in the serine residue. This scenario causes decreased insulin signal transduction (4).

Metabolic diseases related to obesity can change in the gut microbiota composition and function (5). Similarly, the gut microbiota may regulate the development of these metabolic disorders by modulating appetite, energy harvesting and absorption, intestinal barrier function, chronic inflammation, lipid and glucose metabolism, bodyweight gain, and fat storage in hepatic and adipose tissue (6–9).

Studies have shown that obese subjects have less diversity and richness in their gut microbiota than lean subjects (7, 10, 11). Obese subjects also display an increase in *Firmicutes* phylum bacteria which is associated with higher energy absorption from food, and an increase in low-grade inflammation. Consequently, the *Firmicutes/Bacteroidetes* ratio increases with obesity (11), however it is not a consensus (10). An increase in the *Firmicutes/Bacteroidetes* ratio is positively correlated with the development of obesity and insulin resistance (2, 12). It remains unclear whether alterations of the gut microbiota lead to obesity, or weight gain leads to changes in the gut microbiota (11).

In addition to metabolic diseases, diet is an important factor that can modulate the composition and function of the gut microbiota (2, 13, 14). Diets high in fat may reduce microbial diversity and barrier-protecting bacteria, while increasing the abundance of pathogenic bacteria (14–16). By contrast, polyphenols may stimulate beneficial bacteria such as *Lactobacillus* spp. and *Bifidobacterium* spp. in the gut microbiota, and hinder the proliferation of pathogenic strains such as *Clostridium* spp. Polyphenols can also help to control bodyweight by inhibiting appetite, improving lipid metabolism, and inhibiting pancreatic lipase activity (14, 17). The gut microbiota is able to metabolize polyphenols, making them more bioactive, and easily absorbed than the original compounds (7, 13, 18). Both polyphenols and microbiota metabolites may act on metabolic pathways and confer health benefits (19–21). Thus, this review aims to provide an overview of data related to the effect of the two-way interactions of polyphenols and gut microbiota in metabolic disorders.

## Polyphenols: Characterization and Bioavailability

Polyphenols are secondary metabolites of plants, and are widely present in fruit, vegetables, and plant-derived foods such as cocoa, chocolate, tea, coffee, and wine. Polyphenols may influence several metabolic or signaling pathways involved in CVD, T2D, gut health, and cancer (22). Based on their chemical structure and complexity, polyphenols are classified as either flavonoids or non-flavonoids. Flavonoids have several subclasses: flavones, flavanones, flavonols, flavan-3-ols, anthocyanidins, and isoflavones. Non-flavonoid phenolics have a more diverse group of compounds, including phenolic acids, lignans, and stilbenes (22, 23) (Table 1). Many physicochemical factors may affect the bioavailability, such as polarity, molecular mass, plant matrix, digestibility by gastrointestinal enzymes, and absorption on enterocytes and colonocytes. Bio-accessibility is another important factor in bioavailability (22).

**Table 1:** Polyphenols characterization, metabolism, and biological activities (13, 23–26).

**Source**	**Classes**	**Polyphenol**	**General structure**	**Metabolic fates**	**Activities**
**FLAVONOIDS**					
Olive oil, herbs, pistachio, citrus juices, lentils	Flavones	Apigenin Diosmin Luteolin Baicalein Chrysin	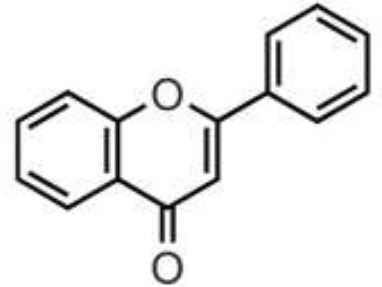	Deglycosylation by the gut microbiota Apigenin aglycone 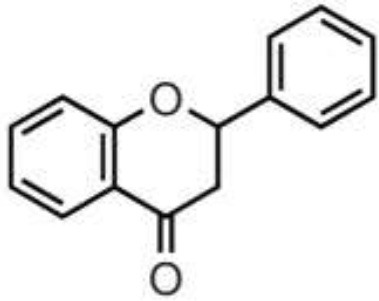 Naringenin 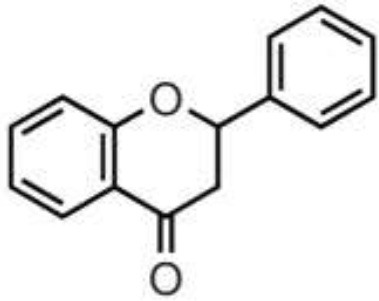 phenylpropionic acids Luteolin: isomerized to eriodyctiol 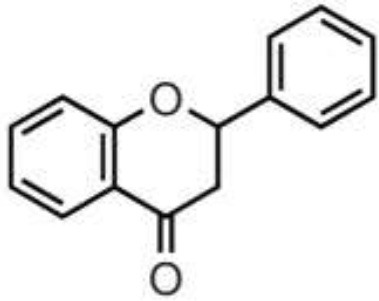 dihydroxyphenyl propionic acid	Anti-inflammatory, antioxidant, regulation of glucose and lipid metabolism, antiviral, antibacterial, anti-parasitic
Citrus fruits, herbs, almond, pistachio	Flavanones	Didymin Eriodyctiol Hesperetin Naringenin	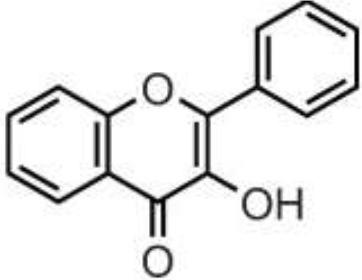	Deglycosylation, dihydroxylation and ring fission by colonic microbiota to phenolic acids	Anti-inflammatory, antioxidant, regulate glucose and lipid metabolism, prevent hepatic steatosis, antifungal, antibacterial, antiviral, antiparasitic
Almond, pistachio, onion, wines, berries, apple, spices, tomato, cocoa, chocolate, citrus fruits	Flavonols	Isorhamnetin Kaempferol Myricetin Quercetin Rhamnetin	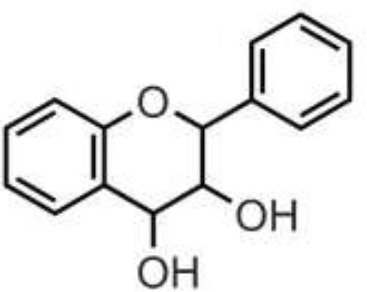	Deglycosylation and phase II metabolism in small intestine; Deglycosylation, dihydroxylation and ring fission by gut microbiota to phenolic acids	Anti-inflammatory, antioxidant, antiviral, antibacterial
Tea, wines, chocolate, cocoa, berries, drupes, tropical, and pomes fruits, herbs, nuts, pulses	Flavan-3-ols	(+)-Catechin (–)-Epicatechin (+)-Gallocatechin (–)-Epigallocatechin (–)-Epigallocatechin gallate Proanthocyanidins	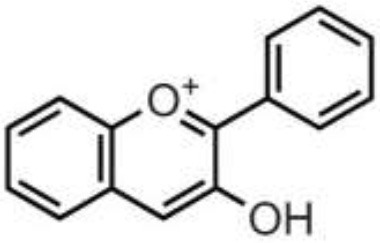	Absorbed in enterocytes without deconjugation or hydrolysis, except Proanthocyanidins	Antibacterial, anticancer, anti-inflammatory, antioxidant, antiviral, antiparasitic
Wine, grape, berries, beans	Anthocyanidins	Cyanidin Delphinidin Malvidin Pelargonidin Peonidin Petunidin	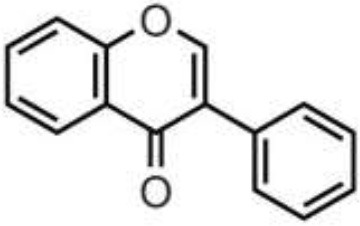	Deglycosylation and phase II metabolism in small intestine; Deglycosylation, dihydroxylation and ring fission by gut microbiota to phenolic acids	Antidiabetes, anti-inflammatory, neuroprotective, antioxidant, antiallergic, antibacterial, anticancer
Soy, lentils, chickpeas, peanut, common bean	Isoflavones	Daidzein Genistein Glycitein Biochanin A Formononetin	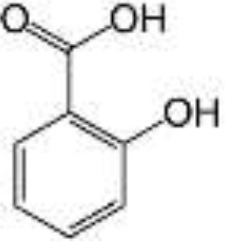	Metabolized by gut microbiota to equol	Estrogenic activity, Anti-inflammatory, hypocholesterolemic effect, antioxidant, antiobesity, antidiabetes
**NON-FLAVONOIDS**					
Coffee, tea, cocoa, drupes, chocolate, walnuts, berries	Phenolic acids	Hydroxybenzoic acids	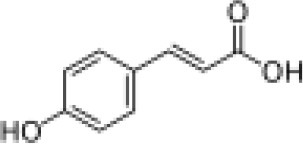	Free structure is absorbed in the small intestine; Esterified to sugars, organic acids, and lipids are metabolized by colonic microbiota	Anti-inflammatory, antioxidant, antibacterial, antiviral, antifungal, antiparasitic
		Hydroxycinnamic acids	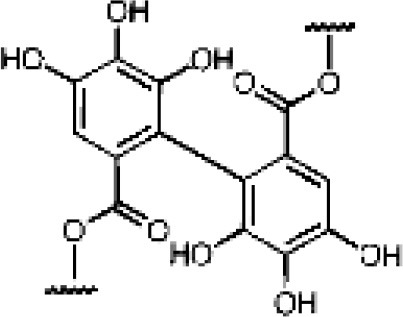		
Pomegranate, grapes, berries, nuts	Hydrolysable tannins	Ellagitannins	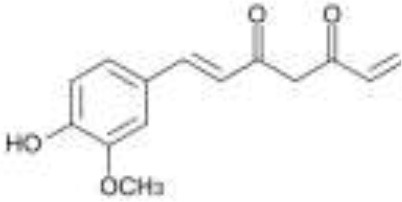	Metabolized by gut microbiota to urolithins	Antioxidant, anticancer, estrogenic activity
Spices	Curcuminoids	Curcumin	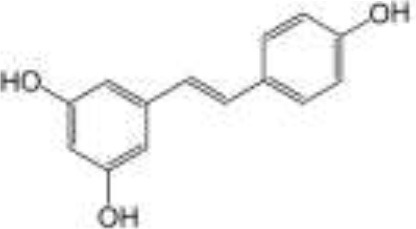	Metabolized by the gut microbiota	Anti-inflammatory, antioxidant, neuroprotective
Wines, chocolate, berries, peanut	Stilbenes	Resveratrol	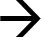	Mainly absorbed in the small intestine and Phase II metabolism; metabolized by gut microbiota to dihydroresveratrol	Anti-inflammatory, antioxidant, cardioprotective, anticancer, antiobesity

Polyphenols present in foods are generally conjugated with sugars or organic acids, or are present as unconjugated oligomers such as condensed tannins. Small amount of the polyphenol's intake (about 5–10%) may be absorbed in the small intestine, mainly those with monomeric, and dimeric structures. The released aglycones enter the enterocyte by passive diffusion. After absorption into the small intestine, aglycones undergo biotransformation in enterocytes and then in hepatocytes. The resultant metabolites are distributed to organs and excreted in the urine. More complex polyphenols, especially oligomeric, and polymeric structures such as condensed or hydrolysable tannins, reach the colon almost unchanged, where they are metabolized by the gut microbiota together with conjugates excreted into the intestinal lumen through the bile. Here, they undergo microbial enzyme transformations, including C-ring cleavage, decarboxylation, dehydroxylation, and demethylation. The result is the generation of less complex compounds such as phenolic acids and hydroxycinnamates (27). Several classes of enzymes—such as α-rhamnosidase, β-glucosidase, and β-glucuronidase—are required to deconjugate specific conjugating moieties. In the case of polymer forms, they are needed to cleave phenolic polymers into individual monomers (28). Once absorbed, polyphenols reach the liver through the portal circulation. Here, they undergo first-pass phase II biotransformation, during which polyphenol aglycones and phenolic acids are conjugated to glucuronides, sulfates and/or methyl-moieties. They then are distributed to organs and excreted in the urine (27).

Oligomeric flavan-3-ols with a degree of polymerization > 3; polymeric flavonols (proanthocyanidins and condensed tannins); esters of hydroxycinnamic acids; and flavonol glucorhamnosides, such as quercetin-3-*O*-glucorhamnoside (rutin) are not absorbed in their original forms. These compounds undergo microbiota transformation on the colon, generating phenolic acids, and other metabolites (29). Proanthocyanidins produce smaller phenolic acids, such as hydroxybenzoic acids, hydroxyphenylacetic acid, hydroxyphenylpropionic acid, hydroxyphenylvaleric acid, or hydroxycinnamic acids, which can be absorbed (30).

Hesperidin and narirutin also pass to the colon, where bacterial enzymes release the aglycone, which is glucuronidated in the intestinal wall. Aglycones can also be metabolized to phenolic acids. Hydroxyphenylpropionic acid and phenylpropionic acid have been described as the main products of naringenin fermentation. Furthermore, 3-(3-hydroxy-4-methoxyphenyl)-propionic acid (dihydroisoferral acid), and various hydroxylated forms of phenylpropionic acid have been reported as colonic catabolites of hesperidin (27).

Ellagitannins undergo intestinal catabolism, possibly generating ellagic acid, which is metabolized by the microbiota into tetra-, tri-, di- and monohydroxyurolithins (24). The bacteria *Gordonibacter urolithinfaciens* and *Gordonibacter pamelaeae* have shown the capacity to biotransformation ellagitannins to urolithins (31).

Resveratrol (3,5,4′-trihydroxystilbene) also reaches the colon. Here, it is subjected to the action of bacteria, which convert it mainly to dihydroresveratrol (3,4-dihydroxystilbene) and lunularin (3,4′-dihydroxybibenzyl) (32).

Studies indicate a two-way interaction between phenolics and gut microbiota. Microbiota may metabolize polyphenols as well as polyphenols and their metabolites may modulate the microbiota by inhibiting pathogenic bacteria and stimulating beneficial bacteria (18, 33–35). Several phenolic compounds have been identified as potential antimicrobial agents with bacteriostatic or bactericidal properties (36). The reciprocal relationship between polyphenols and gut microbiota may contribute to health benefits for the host (34).

## Effects of Gut Microbiota on Polyphenols

The gastrointestinal tract is colonized by several bacterial species, mainly the colon. The mainly microbiota phyla are: *Firmicutes, Bacteroidetes, Proteobacteria, Actinobacteria*, and *Verrucomicrobia* (37). In healthy subjects, *Bacteroidetes* such as *Prevotella* and *Bacteroides* genera, and *Firmicutes* such as *Clostridium, Enterococcus, Lactobacillus*, and *Ruminococcus* genera, represent more than 90% of bacterial species (18, 37). The composition of the individual microbiota varies in certain circumstances, including diarrheal illness and antibiotic therapy, or induced by nutritional intervention (29). Diet strongly influences the gut microbiota and can modify its impact on health, with either beneficial or deleterious consequences. *Prevotella* is the main bacteria in the gut microbial community in people who eat carbohydrate-rich diets, whereas *Bacteroides* is predominant in the gut of people who follow diets rich in animal protein and saturated fat (36, 38). Some bacterias are related to the metabolism of polyphenols, especially *Flavonifractor plautii, Slackia equolifaciens, Slackia isoflavoniconvertens, Adlercreutzia equolifaciens, Eubacterium ramulus, Eggerthella lenta*, and *Bifidobacterium* spp, which participate in the metabolism of several polyphenols (Table 2).

**Table 2:** Polyphenols metabolism by gut microbiota in humans.

**Bacteria**	**Polyphenol Class**	**Bacterial action**	**Main metabolites**	**References**
*Clostridium orbiscidens,* *Eubacterium oxidoreducens,* *Eubacterium ramulus, Eggerthella* sp., *Enterococcus casseliflavus,* *Butyrivibrio* spp., *Flavonifractor plautii, Bacteroides uniformis, Bacteroides ovatus, Bifidobacterium* spp., *Blautia* sp.	Flavonols (Kaempferol Quercetin Myricetin)	*O-*Deglycosylation *C*-ring fission	Protocatechuic acid 2-(3,4-dihydroxyphenyl-acetic acid 2-(4-hydroxyphenil)-propionic acid 3-hydroxyphenylacetic acid, 3-(3-Hydroxyphenyl)-propionic acid 2-(3-hydroxyphenil)-acetic acid 2-(3-dihydroxyphenil)-acetic acid 3-(3,4-dihydroxyphenil)-acetic acid Protocatechuic acid 2-(3,5-dihydroxiphenil)-acetic acid	(26, 38–41)
*Adlercreutzia equolifaciens, Eggerthella* strain Julong732*, Paraeggerthela hongkongensis, Slackia equolifaciens, Slackia isoflavoniconvertens, Clostridium butyricum, Eubacterium ramulus, Flavonifractor plautii*	Flavanones (Naringenin, Hesperidin)	*C*-ring fission	3-(4-hydroxyphenyl)-propionic acid 3-phenylpropionic acid Hydroxyphenylpropionic acid	(39–41)
*Clostridium coccoides, Bifidobacterium* spp*., Eubacterium, Eggerthella lenta, Adlercreutzia equolifaciens, Slackia equolifaciens, Lactobacillus plantarum, Flavonifractor plautii*	Flavan-3-ols Proanthocyanidins	Hydrolysis of ester bonds *C*-ring cleavage Dehydroxylation	3,4-Dihydroxyphenylpropionic acid 3-(3-hydroxyphenyl)-propionic acid 3-Hydroxyphenylpropionic acid 3-Hydroxybenzoic acid 4-Hydroxy-5-(3,4-dihydroxyphenyl) valeric acid 3,4-dihydroxybenzoic acid Phenylvalerolactones	(39–41)
*Lactobacillus plantarum, Lactobacillus casei, Lactobacillus acidophilus, Bifidobacterium lactis*	Anthocyanins Cyanidin, Peonidin, Pelargonidin, Malvidin	*O*-Deglycosylation, *C*-ring fission	3,4-dimethoxybenzoic acid 3,4-dihydroxybenzoic acid 2-(4-hydroxyphenyl)-propionic acid 3-hydroxycinnamic acid 4-hydroxybenzoic acid	(39–41)
*Streptococcus intermedius, Escherichia coli, Bifidobacterium* spp., *Bacteroides ovatus, Streptococcus intermedius*, *Ruminococcus productus, Lactobacillus mucosae, Eubacterium limosum, Enterococcus* *faecium, Enterococcus casseliflavus, Eubacterium ramulus, Veillonella* sp., strain GC19-1, *Eggerthella* Julong 732, *Blautia* sp., *Lactococcus* spp., *Clostridium* spp., *Slackia isoflavoniconvertens, Slackia equolifaciens, Adlercreutzia equolifaciens, Paraeggerthella hongkongensis, Lactobacillus mucosae, Finegoldia magna*	Isoflavones (Daidzein Genistein)	*O*-Deglycosylation *O*-demethylation Reduction *C*-ring cleavage dehydroxylation	Equol *O*-desmethylangolensin (*O*-DMA) 5-hydroxy-equol 2-(4-hydroxyphenyl)-propionic acid Phloroglucinol	(26, 39–41)
*Bacteroides uniformis, Blautia* sp*., Bifidobacterium* spp., *Lactobacillus* spp., *Enterococcus* spp., *Lactococcus lactis, Eubacterium* spp., *Escherichia* sp., *Clostridium butyricum, F. plautii*	Flavones (Luteolin, Apigenin)	*O*-Deglycosylation *O*-Demethylation C-ring fission Dehydroxylation at B-ring	3-(3,4-dihydroxyphenyl)-propionic acid 3-(4-hydroxyphenyl)-propionic acid 3-(3-hydroxyphenyl)-propionic acid 4-hydroxycinnamic acid	(39–41)
*Butyrivibrio* spp., *Gordonibacter urolithinfaciens, Gordonibacter pamelaeae*	Ellagitannins	Hydrolysis	Urolithins	(24, 31, 39)
*Bacteroides distasonis, Bacteroides fragilis, Bacteroides ovatus, Clostridium cocleatum, Clostridium saccharogumia, Butyribacterium methylotrophicum, Eubacterium callanderi, Eubacterium limosum, Blautia producta, Peptostreptococcus productus, Clostridium scindens, Eggerthella lenta, Lactonifactor longoviformis*	Lignans	Deglycosylation Demethylation Dehydroxylation Dehydrogenation	Enterolactone Enterodiol	(39, 40)
*Escherichia coli, Bifidobacterium lactis, Lactobacillus gasseri*	Phenolic acids (Caffeic acid, Ferulic acid)	De-esterification Dehydroxylation	3-(3-hydroxyphenyl)-propionic acid 3-(3,4-dihydroxyphenyl)-propionic acid 3-(4-hydroxyphenyl)-propionic acid Hydroxyphenyl-ethanol Phenylacetic acid Benzoic acid 3-(4-hydroxyphenyl)-propionic acid	(39, 40)
*Slackia equolifaciens, Adlercreutzia equolifaciens*	Resveratrol	Dehydroxylation	Dihydroresveratrol Lunularin	(32)

Interindividual differences in the composition of the gut microbiota may lead to differences in the bioavailability and bioactivity of metabolites. These variations are associated with different metabotypes, which are characterized by the individual's ability to produce specific metabolites (25, 42, 43). *Eubacterium* is related to the metabolism of flavonoids, whereas certain species of the genus *Bifidobacterium* and *Lactobacillus* are involved in the release of hydroxycinnamic acids in the colon (33). *Enterococcus casseliflavus* is involved in the hydrolysis of sugar moieties, such as in quercetin-3-*O*-glucoside, whose process releases the aglycone quercetin and produces lactate, formate, acetate, and ethanol. Moreover, *Eubacterium ramulus, E. oxidoreducens, Flavonifractor plautii* and *Clostridium* strains may metabolize quercetin, leading to the formation of short-chain fatty acids (SCFAs), taxifolin, and 3,4-dihydroxyphenyl-acetic acid (38).

It has been reported that some people produce equol and *O-*desmethylangolensin (*O*-DMA) from isoflavones; they are called “producers.” Others, however, do not produce these metabolites (“non-producers”) (42). This could explain why some subjects did not respond to isoflavone intervention to reduce the symptoms of menopause, once the effect was related to equol*. Adlercreutzia equolifaciens, Eggerthella* strain Julong 732, *Paraeggerthella hongkongensis, Slackia equolifaciens*, and *S. isoflavoniconvertens* have been identified as able to convert isoflavones to equol (39). Different metabotypes are also observed for ellagitannin and ellagic acid, whose metabolism to urolithins in the gut also shows vast human individual variability associated with differences in the colon microbiota. In “urolithin metabotype A,” only urolithin-A conjugates are produced; in “urolithin metabotype B,” isourolithin-A and/or urolithin-B are produced in addition to urolithin-A. In “urolithin metabotype 0,” urolithins are not produced. *Gordonibacter urolithinfaciens* sp. was identified and showed the capacity to convert ellagitannins to urolithins (31, 44).

A recent clinical trial by our group to evaluate the bioavailability and metabolism of anthocyanins and ellagitannins showed that the gut microbiota catabolites both classes of polyphenols. However, some subjects excrete high amounts of polyphenol metabolites in the urine, whereas others excrete low amounts of metabolite. These findings indicate the high interindividual variability regarding polyphenol metabolism (45). Another recent study (43) showed that pomegranate ellagitannins differently improved CVD biomarkers depending on the individual's urolithin metabotypes (UM). The biomarkers were reduction levels of Tchol, small low-density lipoprotein (LDL-c), apolipoprotein (apo) B, oxidized LDL (oxLDL), and non-high-density lipoprotein (HDL-c). That is, CVD risk was reduced only in UM-B subjects who displayed an increase in *Gordonibacter* levels. Despite these examples, the human gut bacteria involved in most dietary polyphenol transformations remain unknown (31).

Weight gain and gut microbiota dysbiosis favor the growth of bacteria that produce isourolithin-A and urolithin-B rather than bacteria that produce urolithin-A (31, 44). Recently, Selma et al. (44) observed that urolithin-A was positively correlated with apo A-1 and intermediate HDL-c, whereas isourolithin-A and urolithin-B were positively correlated with total cholesterol, LDL-c, apo B, very low-density lipoprotein (VLDL-c), intermediate density lipoprotein (IDL-c) and oxLDL. The authors concluded that overweight subjects UM-B were at increased risk of cardiometabolic disease, while those with UM-A had greater protection against cardiometabolic factors.

Oral consumption of a dose of resveratrol (0.5 mg/kg) by healthy subjects showed that dihydroresveratrol (3,4-dihydroxytrans-stilbene) and lunularin (3,4'- dihydroxy-bibenzyl) are the main final metabolites derived from the microbial metabolism of resveratrol. The same study identified two species of bacteria, *Slackia equolifaciens*, and *Adlercreutzia equolifaciens*, related to the production of dihydroresveratrol (32). Just as gut microbiota can affect polyphenol metabolism to generate more bioactive metabolites, polyphenols can also affect the microbiota composition.

## Effects of Polyphenols on Gut Microbiota

The mechanisms by which polyphenols modulate the gut microbiota still need to be elucidated, but may involve both direct and indirect interactions. These compounds can directly stimulate or inhibit bacterial growth. Inhibition refers to the bactericidal or bacteriostatic effect of phenolic compounds, which inhibits the growth of potentially pathogenic bacteria while minimally affecting—or even increasing—the population of beneficial bacteria. However, it is important to consider the concentration and characteristics of these compounds; that is, the type of compound and whether it occurs in conjugated or free form. Indirectly, phenolic metabolites can affect the growth of one group of bacteria by increasing the development of another group (24, 33). Resveratrol presented a relevant antibacterial activity on clinically important bacteria, such as *Salmonella enterica, Enterococcus faecalis*, and *Escherichia coli* (46).

The effects of polyphenols on gut microbiota have been shown *in vitro, in vivo* and in human studies (Table 3).

**Table 3:** Summary of the main findings of studies related to the effects of polyphenols on gut microbiota and metabolic outcomes.

**Model**	**Intervention time**	**Polyphenols**	**Dose**	**Bacteria**	**Metabolic outcomes**	**References**
***In vitro***						
Batch-culture fermentation	—	(+)-catechin	150 mg/mL	↑*Clostridium coccoides* -*Eubacterium rectale* group ↑*Bifidobacterium* spp. ↑*Escherichia coli* ↓*Clostridium histolyticum*	—	(47)
Three-stage culture system	—	Cocoa		↑*Bifidobacteria* ↑*Lactobacilli*	↑ butirate	(48)
Batch-culture fermentation	24 h	malvidin-3-glucoside, gallic acid and a mixture of anthocyanins		↑*Bifidobacterium* spp. ↑*Lactobacillus*	—	(49)
Culture	24 h	Chinese tea extract	0,1%, w/v of polyphenols	↓*Clostridium perfringens* ↓*Clostridium difficile* ↓*Bacteroides* spp.	—	(50)
Fermentation	36 h	Green tea, oolong tea and black tea		↑*Bifidobacterium* ↑*Lactobacillus* spp. ↑*Enterococcus* spp. ↓*Bacteroides* ↓*Prevotella* ↓*Clostridium histolyticum*	↑ SCFAs	(51)
***In vivo***						
C57BL/6J mice	30 days	Resveratrol	4 g/kg bw	↑*Bifidobacterium* ↑*Lactobacillus* ↑*Bacteroides* ↑*Akkermansia* ↓*Prevotella* ↓*Ruminococcaceae* ↓*Anaerotruncus* ↓*Alistipes* ↓*Heliobacter* ↓*Peptococcaceae* ↓ F/B ratio	—	(52)
Obese rats (HFD−45% kcal fat)	10 weeks	QuercetinResveratrol	30 mg/kg bw 15 mg/kg bw	↓ Firmicutes ↓ F/B ratio	↓ bw gain ↓ visceral adipose tissue weight ↓ serum lipids ↓ serum inflammation markers (TNF-α, IL-6, MCP-1) Improved serum adiponectin, insulin and leptin	(53)
Obese C57BL/6J mice (HFD−162 g fat/kg diet)	12 weeks	Blueberry extract	200 mg/kg bw	↑*Proteobacteria* ↑*Bifidobacterium* ↑*Helicobacter* ↓*Actinobacteria* ↓*Prevotella*	↓ bw gain ↓ serum LDL-c ↓ total cholesterol in the liver	(14)
Obese C57BL/6J mice (HFHS diet−32% kcal fat and 25% kcal sucrose)	4 weeks	Decaffeinated green tea Black tea	240 mg/kg bw 320 mg/kg bw	↓ Firmicutes ↑ Bacteroidetes ↑*Pseudobutyrivibrio* (black tea)	↓ bw ↑ SCFAs (black tea)	(54)
Rats	6 weeks	Lowbush wild blueberries	24 mg anthocyanins/day	↓*Lactobacillus* ↓*Enterococcus* ↑*Bifidobacteria* ↑*Coriobacteriacea*	—	(55)
Rats	25 days	Resveratrol	1 mg/kg bw	↑*bifidobacteria* ↑*lactobacilli* ↓*Enterobacteria*	Protection of colonic mucosa architecture ↓ Systemic inflammation biomarkers (IL-6, haptoglobin and fibrinogen)	(56)
Obese C57BL/6J mice (HFHS diet)	8 weeks	Arctic-berry extract		↑*Akkermansia muciniphila* ↑*Oscillibacter* ↑*Turicibacter*	↓ Fasting and postprandial hyperinsulinemia ↓ Circulating endotoxemia ↓ Liver TG depots ↓ Intestinal and hepatic inflammation	(57)
Mice (HFHS diet)	8 weeks	Camu-camu extract	200 mg/kg	↓ F/B ratio ↑ Microbial richness	↓ Bw gain ↓ Fat accumulation ↓ Metabolic inflammation ↓ Endotoxemia ↑ Glucose tolerance ↑ Insulin sensitivity Protected from hepatic steatosis	(21)
Balb/c mice (HFD)	4 weeks	Pomegranate-peel extract	6 mg/day	↑ Caecal pool of *Bifidobacteria*	↓ Serum total cholesterol ↓ LDL-c ↓ Inflammatory markers in colon and visceral adipose tissue	(58)
Rats (HFD−60% kcal fat)	10 weeks	Coffee	20 g/L	↓ F/B ratio ↓*Clostridium leptum* ↑*Enterobacteria*	↓ bw ↓ Adiposity ↓ Livre TG ↓ Energy intake ↓ Insulin resistance	(19)
Obese C57BL/6J mice	16 weeks	Grape		↑*Akkermansia muciniphila* ↑ *Alistipes* spp. ↓ *Clostridiale* ↓ F/B ratio	↑ Glucose tolerance ↓ Metabolic endotoxemia ↓ Intestinal and systemic inflammation ↓ Liver TG	(59)
**CLINICAL TRIALS**						
Overweight obese subjects with mild hyperlipidemia	3 weeks	Pomegranate extract	656 mg of polyphenols	↑*Odoribacter* ↑*Bacteroides* ↑*Faecalibacterium* ↑*Butyricicoccus* ↑*Butyricimonas* ↓*Parvimonas* ↓*Metanobrevibacter* ↓*Metanosphaera*	↓ LBP	(6)
Healthy subjects	4 weeks	Cocoa flavonols	494 mg/day	↑ *Bifidobacterium* ↑ *Lactobacillus* ↓ *Clostridia*	↓ Plasma TG ↓ Plasma CRP	(60)
Subjects with metabolic syndrome	30 days	Dealcoholized red wine	272 mL/day	↑ *Bifidobacterium* ↑*Lactobacillus* ↑*Faecalibacterium prausnitzii* ↑*Roseburia* ↓ F/B ratio	—	(61)
Healthy men	4 weeks	Red wine	272 mL/day	↑*Enterococcus* ↑*Prevotella* ↑*Bacteroides* ↑*Bifidobacterium* ↑*Enterococcus* ↑*Bacteroides uniformis* ↑*Eggerthella lenta* ↑*Blautia coccoides-Eubacterium rectale* group	↓ Blood pressure ↓ TG ↓ Total cholesterol ↓ CRP	(62)
Healthy subjects	7 days	Orange juice	500 mL/day	↑ Mogibacteriaceae ↑ Tissierellaceae ↑ Veillonellaceae ↑ Odoribacteraceae ↑ Ruminococcaceae	—	(63)

*Bw, body weight; CRP, C-reactive protein; F/B, Firmicutes/Bacteroidetes; HFD, high fat diet; HFHS, high fat, high sucrose; IL-6, Interleukin-6; LBP, lipopolysaccharide binding protein; LDL-c, low density lipoprotein; MCP-1, monocyte chemoattractant protein-1; SCFAs, short chain fatty acids; TG, triacylglycerol; TNF-α, tumor necrosis factor alpha; w/v, weight/volume*.

Polyphenols can enhance the abundance of beneficial bacteria such as *Bifidobacterium* and *Lactobacillus* which contribute to the gut barrier protection; *Faecalibacterium prausnitzii* which presents anti-inflammatory action by blocking nuclear factor-kappa B (NF-kB) activation; and *Roseburia* sp. which are butyrate producers (61).

Overall, the polyphenol structure, the dosage evaluated, and the strain of microorganism can influence the effect of polyphenols on bacterial growth and metabolism. In this context, Gram-positive bacteria are more sensitive to polyphenols than are Gram-negative bacteria. This variability might be due to the differences in their wall composition (25).

## Effects of Phenolic-Gut Microbiota Interactions on Obesity and Related Metabolic Diseases

Obesity is characterized by chronic, low-grade inflammation which may have a major role in the initiation and development of metabolic diseases. Low-grade inflammation increases immune system cell infiltration and the production of inflammatory cytokines in adipose tissue. Polyphenols and their bacterial metabolites can act against obesity by modulating the development of adipose tissue and the obesity-induced inflammatory genes (64). It should be highlighted that most polyphenols inhibit the NF-kB pathway and consequently the expression of inflammatory genes possibly by a mechanism involving microRNAs (miRNAs) (35, 64). Polyphenols can modulate more than 100 miRNAs involved in the regulation of different cellular processes such as inflammation and apoptosis (35). In addition, in mice 3T3-L1 adipocytes treated with açaí (*Euterpe oleracea* Martius) extract containing cyanidin-3-rutinoside and cyanidin-3-glucoside, there was a reduction in leptin and plasminogen activator inhibitor-1 (PAI-1) levels and an increase in adiponectin levels. This extract also reduced oxidative stress and inhibited the NF-kB pathway (65). Gonzales and Orlando (66) also observed inhibition of the NF-kB pathway and the inflammatory genes expression when adipocytes were treated with curcumin or resveratrol.

Other potential anti-obesity mechanisms of polyphenols include inhibition of digestive enzymes and consequently reduce energy efficiency, glucose homeostasis improvement, suppression of adipogenesis and lipogenesis, increase of energy expenditure via thermogenesis, and of fat oxidation, and excretion of fecal lipids (20). Resveratrol, for example, can decrease obesity by reduction of *de novo* lipogenesis and adipogenesis, increase of adipocytes apoptosis, and oxidation of fatty acids. Evidence indicates that resveratrol regulates cell-signaling pathways and gene expression (67). In a recent study, overweight and obese subjects consumed 282 mg/day of epigallocatechin gallate (EGCG) and 80 mg/day of resveratrol for 12 weeks. These polyphenols downregulated the expression of genes related to adipogenesis and apoptosis (adipocyte turnover), energy metabolism, oxidative stress and inflammation (68).

Flavonoids can improve glucose homeostasis mainly by the modulation of gene expression that codes key metabolic proteins. These gene modifications can result from the interaction of flavonoids with signaling cascades and/or with epigenetic factors such as miRNAs (69). Polyphenols such as green tea polyphenols, cinnamon, and grape seed proanthocyanidins can delay gastric emptying rate and decrease postprandial feeling of hunger by regulating plasma insulin and glucagon-like peptide (GLP)-1 levels (70, 71). GLP-1 inhibits glucagon secretion by hampering the gluconeogenesis in the liver and thereby improves insulin sensitivity (72). Moreover, polyphenols such as chlorogenic acid and ferulic acid can upregulate the expression of GLUT-4 and peroxisome proliferator-activated receptor (PPAR)-γ improving glucose uptake into the cells (73).

Polyphenols can interact with cell membranes, changing their structure and function. They also can interact with cellular receptors, modulate the activities of enzymes and transcription factors, and affect gene expression. Polyphenols influence molecular signal transduction pathways such as inflammation cascade, cell proliferation/migration, oxidative stress, and metabolic disorders (73). Flavonoids exert anti-inflammatory activity by inhibiting proinflammatory gene expression such as phospholipase A2 (PLA2), cyclooxygenase (COX)-2, lipoxygenase (LOX), or inducible nitric oxide synthase (iNOS) through the PPAR-γ activation; inhibit NF-kB, mitogen-activated protein kinase (MAPK) and c-JUN pathways; and activate phase II antioxidant enzymes, and serine/threonin protein kinase Akt/PKB (74).

Polyphenols are mainly metabolized by the colonic microbiota, forming more bioactive metabolites than those consumed in food. Along with the modulation of the colonic microbiota, polyphenol-derived metabolites may contribute to host health benefits (75). The gut microbiota helps humans to maximize the absorption of nutrients and energy from the diet, and plays an essential role in physical health status. Microbial infections and gut microbiota dysbiosis are associated with metabolic disorders (18).

Gut microbiota has been considered a potential new contributor to the growing prevalence of obesity and associated cardiometabolic disorders, such as metabolic syndrome, inflammation, and T2D (76, 77). Subjects with low bacterial richness show increased dyslipidemia, adiposity, insulin resistance, and inflammatory phenotype (7, 8). Obese subjects transplanted with the microbiota from lean donors presented increased bacterial diversity in their gut, with an associated increase in butyrate-producing bacteria and subsequent increase in insulin sensitivity (78). The same results were observed in animals. The gut microbiota of genetically obese mice (*ob/ob)* harvests more energy than that of their lean *ob/*+ counterparts. This phenotype was transferred in germ-free mice transplanted with the microbiota from obese donors (8, 15). Still, the body-fat mass of germ-free mice increased 60% in 2 weeks after receive the microbiota from conventional mice; this was accompanied by increased levels of circulating glucose and leptin, insulin resistance, and adipocyte hypertrophy. These results can be partly explained by the capacity of the gut microbiota to breakdown undigestible polysaccharides into monosaccharides that could be absorbed, whose fact increased hepatic lipogenesis (10).

It should be noted that the gut microbiota can influence energy metabolism and homeostasis. It does so by regulating the use of energy from the diet, interacting with signaling molecules involved in the metabolism of microorganisms, modifying intestinal permeability, and releasing intestinal hormones—such as peptide YY (PYY) and GLP-1 (79). *Akkermansia muciniphila*, a specie increased by polyphenols, was correlated with increased L-cells, the source of GLP-1 and GLP-2 (80). *A. muciniphila* was also inversely linked to visceral fat accumulation, adipocyte size in subcutaneous adipose tissue, and fasting plasma glucose levels in obese humans (10).

Recent studies have indicated that the gut microbiota produces several metabolites, some of which enter systemic circulation and show biological activity. The microbiota, through these bioactive metabolites, can act directly or indirectly in organs, with beneficial or adverse effects. Some of the metabolites—such as SCFAs (acetate, propionate, and butyrate)—may interact with hormones such as ghrelin, leptin, GLP-1, and PYY, which are known to increase satiety and thus reduce bodyweight (46, 81). During high-fat diet (HFD) feeding, the microbiota increases gut permeability through mechanisms that involve GLP-1. The result is systemic inflammation, which induces central inflammation via humoral, cellular (microglial), or unknown neural pathways. Energy homeostasis is thus impaired and food intake continues to increase.

Moreover, SCFAs, mainly butyrate, are used as an energy source for colonocytes. Also, SCFAs can contribute to several metabolic pathways, including gluconeogenesis (propionate) and lipogenesis (acetate) (8). *Firmicutes* are the main butyrate-producing bacteria in the human gut, especially *Clostridium leptum, Faecalibacterium prausnitzii, Roseburia* spp. and *Eubacterium rectale*. In addition, propionate and acetate are mostly produced by the *Bacteroidetes* phylum (77). SCFAs can act as signaling molecules and activate several pathways. An example is the activation of the 5' adenosine monophosphate-activated protein kinase (AMPK) in muscle tissues and in the liver. AMPK activates key factors involved in lipid and glucose metabolism such as PPARγ, PPARγ coactivator 1 alpha (PGC-1α), and liver X receptors (LXR) (77). AMPK is a sensor of adenine nucleotides that is activated in states of low cellular energy. In this context, AMPK can stimulate fatty-acid oxidation and mitochondrial biogenesis, which are alternative mechanisms to generate adenosine triphosphate (ATP) (10, 15). SCFAs may also act as ligands for G-protein-coupled receptors, also called free fatty-acid receptors (GPR or FFAR), in the gut. These are GPR41 (FFAR3), GPR43 (FFAR2), and GPR109A. The result is the suppression of pro-inflammatory cytokine secretion. SCFAs link GPR-41 and GPR-43, stimulating the secretion of GLP-1 and PYY (8, 10, 13). The interaction of butyrate with GPR109A reduces the inflammation mediated by interleukin (IL)-8 and IL-10 and promotes lipolysis in adipose tissue (82). In addition, butyrate may induce fatty-acid oxidation, lipolysis, and thermogenesis, while acetate exerts an anti-lipolytic effect in the WAT, reduces fat accumulation and stimulates mitochondrial activity in the liver (8). The antilipolytic effect of acetate might be caused by reduced phosphorylation of hormone-sensitive lipase in a GPR-dependent manner (9).

AMPK activation induces PGC-1 and SIRT1 expression, which regulates lipolysis-involved cell energy metabolism, and suppresses sterol regulatory element-binding protein 1 (SREBP-1) expression, which regulates the genes involved in lipogenesis required for glucose metabolism and fatty acid and lipid production (16). In this context, blueberry polyphenols increased AMPK phosphorylation in liver and WAT and reduced the expression of genes linked to lipogenesis regulation (PPARγ, FAS, and SREBP-1) in the liver of mice (14). Resveratrol also decreased the expression of FAS, SREBP-1, and SCD-1 while increased the expression of genes involved in glucose and fatty acids oxidation (carnitine palmitoyltransferase (CPT)-1α, pyruvate dehydrogenase kinase (PDK) 4 and PPAR-α) in obese mice (83). In male Zucker (fa/fa) rats, resveratrol supplemented orally (15 mg/kg body weight/d) for 6 weeks, reduced serum TNF-α, monocyte chemoattractant protein-1 (MCP-1), and C-reactive protein (CRP) levels, as well as protein expression of IL-6, the activity of NF-κB and macrophage infiltration in adipose tissue were decreased by resveratrol (84).

**Figure 1:**
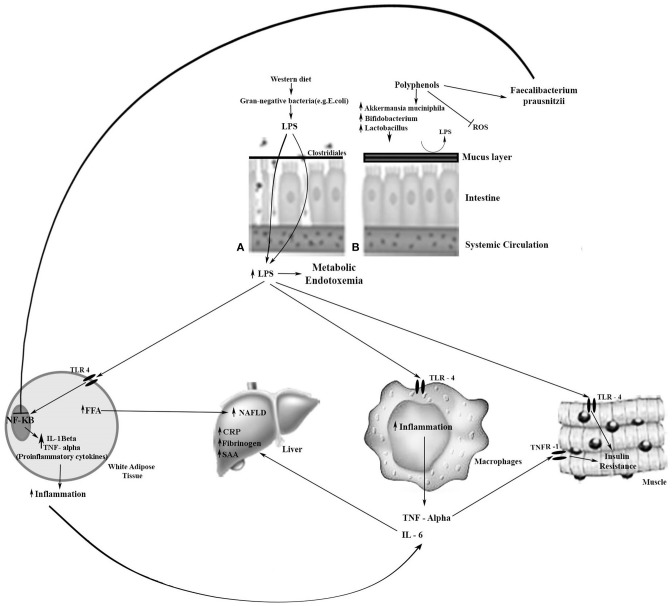
Metabolic effects of LPS and the effect of polyphenols on beneficial bacteria on enterocytes. **(A)** Leaky and inflamed gut. A Western diet, rich in simple carbohydrates (sugar), saturated fatty acids, and low in dietetic fiber, may cause obesity-related dysbiosis, and, consequently, loss of gut barrier integrity. The reduction of mucosal layer thickness and increase in the gut barrier permeability favors the LPS pass through the intestinal cells to the bloodstream, resulting in metabolic endotoxemia. LPS is transported to the target tissues by chylomicrons. LPS binds to TLR-4 in the target tissue and triggers an inflammatory response. **(B)** Normal gut. Dietary polyphenols may sequester reactive oxygen species (ROS); increase *Bifidobacterium* spp., *Lactobacillus* spp., and *Akkermansia muciniphila* which are associated with the preservation of the integrity of the intestinal mucus layer and intestinal barrier function; and increase *Faecalibacterium prausnitzii* which inhibits the NF-kB activation. Thereby, there is a reduction in lipid storage, insulin resistance, and inflammation. →, Activation; ⊣, Inhibition; CRP, C-reactive protein; FFA, free fatty acids; IL, Interleukin; LPS, lipopolysaccharide; NAFLD, non-alcoholic fatty liver disease; NF-kB, nuclear factor-kappa B; SAA, serum amyloid A; TLR-4, Toll-like receptor-4; TNF-α, tumor necrosis factor alpha; TNFR-1, TNF receptor-1.

In gut dysbiosis, lipopolysaccharide (LPS), a main component of the outer membrane of Gram-negative bacteria, promotes macrophage recruitment and polarization in WAT, inducing inflammation through the Toll-like receptor (TLR) 4 (Figure 1). LPS binds TLR4 and triggers a cascade of reactions inside the cell that culminates in the release of the NF-kB from the IKK complex and its translocation to the nucleus, activating the inflammatory response. Increase in plasma LPS levels leads to increased gut permeability, probably due to reduced expression of proteins that compose the tight junction, i.e., zonulin and occludin. This proteins create a gut epithelial barrier that prevents the bacterial population and products from the gut lumen reaching the blood circulation (15). High LPS concentration in the circulation causes metabolic endotoxemia and induces the production of inflammatory cytokines and mediators such as CRP, which contribute to chronic low-grade inflammation and subsequent cardiovascular risk increase (6).

LPS is also linked to impairing pancreatic β-cells by suppressing insulin secretion (77). Metabolic endotoxemia is associated with increased body fat, insulin resistance and increased expression of pro-inflammatory biomarkers (85). Therefore, gut microbiota dysbiosis is involved in various chronic conditions, such as obesity, diabetes, metabolic syndrome, and CVD. Dysbiosis might also promote the synthesis of SCFAs, which affect the production of cholesterol and fatty acids in the liver, thereby altering the metabolism of lipids (76). Diet has a fundamental influence on the remodeling of the gut microbiota, altering its composition and functionality, which in turn can modulate the susceptibility to disease (86, 87). Butyrate induces mucus production, which decreases bacterial transport across the epithelium. It also improves gut integrity by increasing tight junction protein expression (15). Propionate increases intestinal gluconeogenesis (88), inhibiting the synthesis of hepatic cholesterol; it may also attenuate the secretion of cytokines (IL-4, IL-10, TNF) and chemokines (9). Polyphenols such as resveratrol can increase the *Faecalibacterium prausnitzii*, which inhibits the NF-kB activation. Curcumin supplementation attenuated the Western-diet-induced increase in plasma LPS levels and improved intestinal barrier function in LDLR^−/−^ mice (89).

The greater density of *Bacteroidetes* has been associated with increased butyrate and propionate levels, which contribute to healthy bodyweight by inhibiting hunger and helping to maintain glucose homeostasis. Both propionate and succinate were described as efficient substrates for glucose production in the liver (7). Human evidence of a beneficial effect of SCFAs on bodyweight control, inflammation, and insulin sensitivity is increasing, as is evidence regarding its role in glucose and lipid homeostasis (9). The health properties attributed to beneficial bacteria (*Bifidobacterium* spp. and *Lactobacillus* spp.) for human hosts are manifold. They include nutrient processing, reduction of serum cholesterol, protection against gastrointestinal disorders and pathogens, reinforcement of intestinal epithelial cell-tight junctions, and increased mucus secretion and modulation of the intestinal immune response through cytokine stimulus (25, 34, 49). The wide diversity of microbial communities among people can result in vast variability in the composition and functions of the interindividual microbiome (24, 76, 86, 87, 90).

Polyphenols have been compared to prebiotics, since by definition prebiotics are non-digestible polysaccharides. After metabolism by gut microbiota, these polysaccharides modulate the composition and/or function of the microbiota, providing a beneficial physiological effect on the host (14, 91). Polyphenols may protect against diet-induced obesity, although their effects on food intake are controversial. Possibly, polyphenols increase the secretion of mucin and remove reactive oxygen species (ROS), creating a beneficial environment for the bloom of the anaerobic *Akkermansia muciniphila*, and ameliorating metabolic endotoxemia (91). Resveratrol exerts effects on intestinal barrier function and integrity. Evidence indicates that resveratrol can upregulate the expression of intestinal tight junction proteins (46). *A. muciniphila, Lactobacillus* spp. and *Bifidobacterium* spp. preserve the integrity of the intestinal mucus and intestinal barrier function, and counteract the deleterious effect of HFD on gut permeability. *A. muciniphila* abundance is inversely correlated with bodyweight and with an improved metabolic profile (7, 13). The abundance of *A. muciniphila* was found to decrease in obese and diabetic animals and humans (7). Treatment with *A. muciniphila* has been suggested to reduce the risk of obesity and related metabolic disorders, because these bacteria have been shown in mice to reverse endotoxemia, inflammation in adipose tissue, gain of adipose mass, and insulin resistance (92).

Phenolic metabolites derived from microbial metabolism may exert an anti-inflammatory effect in human health. Dehydroxylated phenolic acids derived from microbial metabolism of proanthocyanidins reduced the secretion of IL-6, IL-1β, and TNF-α in LPS-stimulated peripheral blood mononuclear cells from healthy subjects (93). Tucsek et al. (94) treated macrophages with LPS to induce an inflammatory response. The authors found that polyphenol metabolites, such as ferulaldehyde, induced an anti-inflammatory response by reducing MAPK activation, which inhibited NF-κB and ROS production. Flavonols and proanthocyanidins provided as cranberry extract attenuated HFD-induced obesity and associated metabolic changes. In addition, the extract increased *Akkermansia muciniphila*, similar to prebiotic administration (80).

Branched-chain amino acids (BCAA) have been shown to be increased in obesity and T2D, contributing to the development of obesity-related insulin resistance. Possibly, polyphenols from blueberry powder can increase genes for BCAA degradation and consequently improve insulin sensitivity (16).

The beneficial effects of polyphenols in humans are still inconclusive. One reason for that is the high interindividual variation related to polyphenols metabolism and the heterogeneity of individual biological responsiveness to their intake. Most evidence of the anti-obesity effect of polyphenols comes from animal studies (14, 16, 21, 83). Whether animal findings can be extrapolated to humans still warrants further investigation. Moreover, several *in vitro* studies have used dietetic polyphenols instead of the bioactive metabolites. Often polyphenols are used at levels above the physiological concentration. It is necessary to establish the concentration of polyphenols in the circulation and tissues, and to perform cell studies using physiological concentrations of the bioactive metabolites. In addition, more well-designed clinical trials that consider interindividual variation in polyphenol metabolism, and the key role of the microbiota are needed to establish the role of polyphenols in obesity-related metabolic diseases. The association of the microbiome analysis with other omics such as genomics, transcriptomics, proteomics, and metabolomics will be able to clarify the biological effects of the polyphenol-microbiota interactions.

## Concluding Remarks and Future Perspectives

Evidence highlights the importance of the gut microbiota in metabolic diseases such as obesity and T2D, by affecting key pathways such as energy homeostasis and inflammation. Diet plays a prominent role in modulating the gut microbiota. The two-way interactions between polyphenols and the gut microbiota may contribute to host health benefits. This two-way interaction entails microbial degradation of polyphenols and modulation of gut microbiota by polyphenols and their metabolites, which inhibits pathogenic bacteria and stimulates beneficial bacteria.

Despite the possible benefits of polyphenols for human health through modulating the microbiome, studies have been scarce and present several limitations. The human gut microbiome composition is complex, and the relative proportion of bacteria types varies widely among individuals. In addition, the metabolome is influenced not only by the gut microbiota but also by genetic and environmental factors. Hence, significant differences in metabolite concentrations may be observed even if subjects consume the same diet. Thus, an effective association of metagenomics with other “omics” data can clarify the complex biosynthetic pathways of the gut microbiota. Insight obtained from such integrative studies may also simplify the development of non-invasive diagnostic tools for preventing and treating metabolic diseases, and help to optimize personalized medicine.

## Author Contributions

TC and MR designed the review. TC wrote the manuscript. MR, NH, and FL revised and approved the manuscript.

### Conflict of Interest

The authors declare that the research was conducted in the absence of any commercial or financial relationships that could be construed as a potential conflict of interest.
